# REW-ISA V2: A Biclustering Method Fusing Homologous Information for Analyzing and Mining Epi-Transcriptome Data

**DOI:** 10.3389/fgene.2021.654820

**Published:** 2021-05-28

**Authors:** Lin Zhang, Shutao Chen, Jiani Ma, Zhaoyang Liu, Hui Liu

**Affiliations:** ^1^Engineering Research Center of Intelligent Control for Underground Space, China University of Mining and Technology, Ministry of Education, Xuzhou, China; ^2^School of Information and Control Engineering, China University of Mining and Technology, Xuzhou, China

**Keywords:** m^6^A methylation, homologous information, iterative signature algorithm, biclustering, unsupervised learning

## Abstract

**Background:** Previous studies have shown that N6-methyladenosine (m^6^A) is related to many life processes and physiological and pathological phenomena. However, the specific regulatory mechanism of m^6^A sites at the systematic level is not clear. Therefore, mining the RNA co-methylation patterns in the epi-transcriptome data is expected to explain the specific regulation mechanism of m^6^A.

**Methods:** Considering that the epi-transcriptome data contains homologous information (the genes corresponding to the m^6^A sites and the cell lines corresponding to the experimental conditions), rational use of this information will help reveal the regulatory mechanism of m^6^A. Therefore, based on the RNA expression weighted iterative signature algorithm (REW-ISA), we have fused homologous information and developed the REW-ISA V2 algorithm.

**Results:** Then, REW-ISA V2 was applied in the MERIP-seq data to find potential local function blocks (LFBs), where sites are hyper-methylated simultaneously across the specific conditions. Finally, REW-ISA V2 obtained fifteen LFBs. Compared with the most advanced biclustering algorithm, the LFBs obtained by REW-ISA V2 have more significant biological significance. Further biological analysis showed that these LFBs were highly correlated with some signal pathways and m^6^A methyltransferase.

**Conclusion:** REW-ISA V2 fuses homologous information to mine co-methylation patterns in the epi-transcriptome data, in which sites are co-methylated under specific conditions.

## Introduction

At present, researchers have identified more than 170 different chemical modifications in RNA (Frye et al., 2018). *N*^6^-methyladenine (m^6^A) is the most common and abundant post-transcriptional RNA modification in mRNAs and long non-coding RNAs (Fu et al., 2014), and its methylation occurs at the sixth position of nitrogen atoms of adenosine. Studies have shown that m^6^A is involved in some RNA metabolic processes such as mRNA transcription, translation, nucleation, splicing and degradation (Ping et al., 2014; Lin et al., 2016; Deng et al., 2018). Besides, m^6^A also plays an important role in the early development of eukaryotic cells, sex determination, antiviral immunity, brain development, and directed differentiation of hematopoietic stem cells (Zhang et al., 2017, 2019a). In addition to the above biological processes, m^6^A modification is also related to many pathological phenomena, such as leukemia, glioma and hepatocellular carcinoma (Lachén-Montes et al., 2016; Chai et al., 2019).

The m^6^A methylation in RNA is a dynamic and reversible process regulated by methyltransferases and demethylases. Since the main role of m^6^A methyltransferases is to catalyze RNA to produce m^6^A methylation modifications, these enzymes are often called “writers.” The most common m^6^A writer is composed of core components METTL3, METTL14, WTAP, and other subunits (Liu et al., 2014; Ping et al., 2014). On the contrary, m^6^A demethylases mainly mediate m^6^A demethylation, so these enzymes are also known as “erasers.” The common erasers are FTO, AKLBH5, and so on (Jia et al., 2011). Studies have shown that m^6^A has a series of biological functions because many RNA binding proteins mediate it. These binding proteins can specifically recognize m^6^A methylated adenosine on RNA, so these proteins are often referred to as “readers.” The common readers include protein YT521-B homologous (YTH) domain family (Meyer et al., 2015), etc. In recent years, with the development of methylated RNA immunoprecipitation sequencing (MeRIP-seq, or m^6^A-seq) technology (Dominissini et al., 2012; Meng et al., 2014), many m^6^A experimental data continue to emerge, which makes it possible to analyze m^6^A in the whole transcriptome. However, since there are a few enzymes, such as m^6^A writers, erasers and readers only, each enzyme may regulate a large number of m^6^A sites. In other words, the methylation level of m^6^A site regulated by the same enzyme may share the same pattern, which is called the co-methylation pattern of m^6^A.

Till this day, some researchers have used clustering methods to study the co-methylation patterns in epi-transcriptome data, trying to clarify the functional mechanism of m^6^A methylation. Based on MeRIP-seq data, Liu et al. used *k*-means clustering, hierarchical clustering, Bayesian factor regression model and non-negative matrix decomposition to cluster m^6^A sites (Liu et al., 2015). To better fit the distribution of epi-transcriptome data, Zhang et al. proposed an infinite beta binomial mixture model based on Dirichlet Process (DPBBM) to reveal the co-methylation patterns (Zhang et al., 2019b). Besides, our previously proposed RNA Expression Weighted Iterative Signature Algorithm (REW-ISA) (Zhang et al., 2020) applied biclustering to the analysis of epi-transcriptome data for the first time. However, the above methods only used the read counts of the m^6^A sites of the IP sample and the input sample in MeRIP-seq data. They did not fully consider the homologous information of sites and experimental conditions. Homology is a central concept in comparative biology, in which the most basic meaning of homology is to have a common ancestor. The homologous information of MeRIP-seq data can be divided into two categories: the genes corresponding to the m^6^A sites and the cell lines (or environments) corresponding to the experimental conditions. Appropriate use of the above-mentioned homologous information will help discover potential local functional blocks (LFBs) and better reveal the m^6^A regulatory mechanism. Besides, although some of the most advanced biclustering methods have been developed, such as runibic (Wang et al., 2016; Orzechowski et al., 2018a), EBIC (Orzechowski et al., 2018b), QUBIC2 (Xie et al., 2020) and RecBic (Liu et al., 2020), their goal is to identify the trend-preserving biclusters. However, when mining m^6^A co-methylation pattern, it is expected to obtain locally hyper-methylated biclusters, so these new methods are not applicable.

Therefore, we proposed an improved RNA expression weighted iterative signature algorithm (REW-ISA V2), which fuses the homologous information of sites and experimental conditions in the iterative search for LFBs. Consistent with the previous method, each potential LFB is identified by the row threshold (defined as *T*_*R*_) and column threshold (defined as *T*_*C*_) during the LFB searching strategy. It is important to note that REW-ISA V2 updates *T*_*R*_ and *T*_*C*_'s selection process, optimizing the selection of thresholds through the built-in rich constraint framework. According to the previous study (Henriques et al., 2015, 2017), REW-ISA V2 is a non-deterministic greedy algorithm, which can be used to find hyper-methylated biclusters. Besides, REW-ISA V2 can obtain these overlapping LFBs when there is overlap between the LFBs implied in the input data.

To verify the effectiveness of the fusion of homologous information, REW-ISA V2 was applied to the collected MERIP-seq data to find potential LFBs. The obtained LFBs were further analyzed by the Gene Ontology (GO) analysis, Kyoto Encyclopedia of Genes and Genomes (KEGG) pathway analysis, and enzyme-specific experiments, in an attempt to reveal the possible regulatory mechanism of m^6^A. As a result, REW-ISA V2 can better find potential LFBs with high methylation levels in the epi-transcriptome data.

## Methods

### Pre-processing of Real Data

As is known, MeRIP-Seq data profiles the m^6^A epi-transcriptome by IP and input samples. Thus, we first need to follow (Chen et al., 2019) and (Wu et al., 2019) to quantify the information of m^6^A sites. Specifically, after downloading the sequencing data from Gene Expression Omnibus (GEO) in SRA format, the Tophat2 (Kim et al., 2013) needs to be used to compare the sequencing data reads with the human reference genome, and finally obtain the Fragments Per Kilobase of transcript per Million (FPKM) statistics.

To mine the potential LFBs in the epi-transcriptome data, only the FPKM statistical information of IP and input samples are not enough. It is necessary to calculate the m^6^A methylation level of each m^6^A site under each experimental condition. Let *m* denote the total number of m^6^A sites and *n* denote the total number of conditions. Therefore, according to the REW-ISA, the methylation level matrix *P* ∈ ℝ^*m*×*n*^ and the RNA expression level matrix *W* ∈ ℝ^*m*×*n*^ can be further calculated using the IP sample and the input samples, as shown in (1, 2).

(1) $$\begin{array}{lll} p_{ij} & = & \frac{t_{ij}\text{+}\alpha }{t_{ij}+h_{ij}\text{+}2\alpha }, \end{array}$$

(2) $$\begin{array}{lll} w_{ij} & = & \log\left(t_{ij}+h_{ij}+1\right). \end{array}$$

In (1) and (2), *t*_*ij*_ represents the FPKM of the *i*-th m^6^A site under the *j*-th condition in the IP sample, and *h*_*ij*_ represents the FPKM of the *i*-th m^6^A site under the *j*-th condition in the input sample. Besides, α in (1) is a very small value, aiming to avoid *NaN* where FPKM of both IP and input samples are zeros. The purpose of introducing the RNA expression level is to provide a confidence level for m^6^A methylation level in further biclustering analysis.

### REW-ISA V2

To eliminate the effect of global sites or conditions on ***P***, REW-ISA V2 performs standard normalization on the whole, rows and columns of ***P*** in turn to eliminate the global effect, as shown in (3–5). ***P***^***nw***^, ***P***^***nr***^, and ***P***^***nc***^ represent the matrices obtained after whole normalization, row normalization, and column normalization, respectively.

(3) $$\begin{array}{l} p_{ij}^{nw}=\frac{p_{ij}-\mathrm{mean}\left(P\right)}{\max\left(P\right)-\min\left(P\right)}, \end{array}$$

(4) $$\begin{array}{l} p_{ij}^{nr}=\frac{P_{ij}^{nw}-\text{mean}(P_{i\cdot }^{nw})}{\max(P_{i.}^{nw})-\min(P_{i.}^{nw})}, \end{array}$$

(5) $$\begin{array}{l} p_{ij}^{nc}=\frac{P_{ij}^{nr}-\text{mean}(P_{.j}^{nr})}{\max(P_{.j}^{nr})-\min(P_{.j}^{nr})}. \end{array}$$

In (3–5), mean(**·**) represents calculating the mean value, max(**·**) represents calculating the maximum value, and min(**·**) represents calculating the minimum value. $P_{i\cdot }^{nw}$ represents the *i*-th row in ***P***^***nw***^, and $P_{\cdot j}^{nr}$ represents the *j*-th column in ***P***^***nr***^. Then min-max normalization is performed on the overall data to generate ***P***^***t***^, which will facilitate subsequent combination with RNA expression level.

(6) $$\begin{array}{l} p_{ij}^{t}=\frac{p_{ij}^{nc}-\min\left(P^{nc}\right)}{\max\left(P^{nc}\right)-\min\left(P^{nc}\right)}. \end{array}$$

For the RNA expression level matrix ***W***, since its distribution fluctuates with the MeRIP-seq data, it is necessary to perform the min-max normalization on ***W*** to generate ***W***^***t***^, which acts as confidence matrix for ***P***^***t***^.

(7) $$\begin{array}{l} w_{ij}^{t}=\frac{w_{ij}-\min\left(W\right)}{\max\left(W\right)-\min\left(W\right)}. \end{array}$$

Suppose that *k*-1 (2 ≤ *k* ≤ *K*) LFBs have been found, and the *k*-th LFB is currently being searched. Assuming that the *k*-th LFB is ***B***_***k***_, the site indicator ρ_*k*_ and the condition indicator κ_*k*_ are used to indicate the sites and conditions contained in ***B***_***k***_. Specifically, the site indication ρ_*ik*_ is one if the *i*-th site is present in ***B***_***k***_ (zero otherwise). The condition indication κ_*jk*_ is one if the *j*-th condition is present in ***B***_***k***_ (zero otherwise). The average methylation level $\mu_{k}^{p}$ and average expression level $\mu_{k}^{w}$ of ***B***_***k***_ can be further calculated, as shown in (8, 9), respectively.

(8) $$\begin{array}{l} \mu_{k}^{p}=\frac{\sum_{i=1}^{m}\sum_{j=1}^{n}p_{ij}^{t}\rho_{ik}\kappa_{jk}}{\sum_{i=1}^{m}\rho_{ik}\sum_{j=1}^{n}\kappa_{jk}}, \end{array}$$

(9) $$\begin{array}{l} \mu_{k}^{w}=\frac{\sum_{i=1}^{m}\sum_{j=1}^{n}w_{ij}^{t}\rho_{ik}\kappa_{jk}}{\sum_{i=1}^{m}\rho_{ik}\sum_{j=1}^{n}\kappa_{jk}}. \end{array}$$

Each time a LFB is found, the average methylation level and average expression level of the LFB should to be removed from ***P***^***t***^ and ***W***^***t***^. The purpose of removing is to prevent the algorithm from falling into a loop looking for a strong LFB. Let residual matrix ***P***^**(*k*)**^ represent the methylation level matrix after eliminating the μ^*p*^ of the first *k*-1 LFBs,

(10) $$\begin{array}{l} p_{ij}^{\left(k\right)}=p_{ij}^{t}-\sum_{z=1}^{k-1}\left(\mu_{z}^{p}\rho_{iz}\kappa_{jz}\right). \end{array}$$

Then, ***P***^**(*k*)**^ turns into ***P***^***R***(***k*****)**^ after row min-max normalization and turns into ***P***^***C***(***k*****)**^ after column min-max normalization. Similarly, let ***W***^**(*k*)**^ represent the RNA expression level matrix after eliminating the μ^*w*^ of the first *k*-1 LFBs,

(11) $$\begin{array}{l} w_{ij}^{\left(k\right)}=w_{ij}^{t}-\sum_{z=1}^{k-1}\left(\mu_{z}^{w}\rho_{iz}\kappa_{jz}\right). \end{array}$$

After obtaining the above ***P***^***R***(***k*****)**^, ***P***^***C***(***k*****)**^ and ***W***^**(*k*)**^, combined with the homologous information of sites and conditions, the algorithm begins to search for LFBs iteratively. The algorithm running from a randomly selected site's subset ***U*****′** and updates the conditions' subset ***V*****′** according to (12).

(12) $$\begin{array}{l} \{\begin{array}{l} e_{{\text{U}}^{\prime }v}^{C}\;=\frac{1}{\left|U^{\prime }\right|}\sum u\;\in \;{\text{U}}^{\prime }\;(w_{uv}^{\left(k\right)}\;\cdot \;p_{uv}^{R(k)})\;v\;\in \;V\\ t_{{\text{U}}^{\prime }v}^{C}=\left|\rho (P_{U^{\prime }\;v}^{t}\cdot W_{U^{\prime }\;v}^{t},\;\frac{\sum_{b\in H_{v}^{C}}\left(P_{{\text{U}}^{\prime }\;b}^{t}\cdot W_{{\text{U}}^{\prime }b}^{t}\right)}{\left|H_{v}^{C}\right|})\right|v\;\in \;H_{v}^{C},\;H_{v}^{C}\in \;V,\\ V^{\prime }=\{v\in \;V:\left|e_{{\text{U}}^{\prime }\;v}^{C}\cdot t_{{\text{U}}^{\prime }\;v}^{C}-\frac{1}{\left|\;V\right|}\sum_{v\in V}e_{{\text{U}}^{\prime }\;v}^{C}\cdot t_{{\text{U}}^{\prime }\;v}^{C}\right|\text{ ​}>\text{ ​}\frac{T_{C}}{\sqrt{\left|U^{\prime }\right|}}\}\; \end{array} \end{array}$$

where ***V*** is the conditions set of ***P***^***t***^, refers to the *u*-th site under the *v*-th condition in ***P***^***R***^, is the RNA expression level of the *u*-th site under *v*-th condition, $H_{v}^{C}$ represents the subset of homologous conditions corresponding to the *v*-th condition. ρ(·) represents to calculate Pearson similarity, |·| represents to calculate absolute value (or module). Besides, ***T***_***C***_ is a hyperparameter, and its function is to select the subset of conditions ***V*****′**. In (12), $e_{{\text{U}}^{\prime }v}^{C}$ is calculated based on ***P***^*R*(***k***)^ and ***W***^(***k***)^, which represents the average methylation level score of the *v*-th condition combined with the confidence of the expression level. $t_{U^{\prime }v}^{C}$ is calculated based on ***P***^***t***^ and ***W***^***t***^, representing the average similarity score of the *v*-th condition relative to its homologous conditions subset. In the process of calculating $e_{U^{\prime }v}^{C}$ and, only the sites involved in ***U′*** are considered.

Then, the subsets of sites are updated following (13).

(13) $$\begin{array}{l} \{\begin{array}{l} e_{u\;V^{\prime }}^{R}=\frac{1}{\left|V^{\prime }\right|}\sum_{v\in V^{\prime }}\left(w_{uv}^{\left(k\right)}\cdot p_{uv}^{C(k)}\right)\;u\in \;U\\ t_{uV^{\prime }}^{R}=\left|\rho (P_{u\;V^{\prime }}^{t}\cdot W_{u\;V^{\prime }}^{t},\frac{\sum_{a\in H_{u}^{R}}\left(P_{a\;V^{\prime }}^{t}\cdot W_{a\;V^{\prime }}^{t}\right)}{\left|H_{u}^{R}\right|})\right|u\in H_{u}^{R},H_{u}^{R}\in U,\\ U^{\prime }=\{u\in \;U:\left|e_{u\;V^{\prime }}^{R}\cdot t_{u\;V^{\prime }}^{R}-\frac{1}{\left|\;U\right|}\sum_{u\in U}e_{u\;V^{\prime }}^{R}\cdot t_{u\;V^{\prime }}^{R}\right|>\frac{T_{R}}{\sqrt{\left|V^{\prime }\right|}}\} \end{array} \end{array}$$

where ***U*** is the sites set of ***P***^***t***^, refers to the *u*-th site under the *v*-th condition in ***P***^***C***^, $H_{u}^{R}$ represents the subset of homologous sites corresponding to the *u*-th site. Besides, *T*_*R*_ is a hyperparameter, and its function is to update the subset of sites ***U′***. $e_{u{\text{V}}^{\prime }}^{R}$ represents the average methylation level score of the *u*-th site combined with the confidence of the expression level. $t_{uV^{\prime }}^{R}$ represents the average similarity score of the *u*-th site relative to its homologous sites subset. In the process of calculating $e_{uV^{\prime }}^{R}$ and $t_{uV^{\prime }}^{R}$, only the conditions involved in ***V′*** are considered.

**Figure 1:**
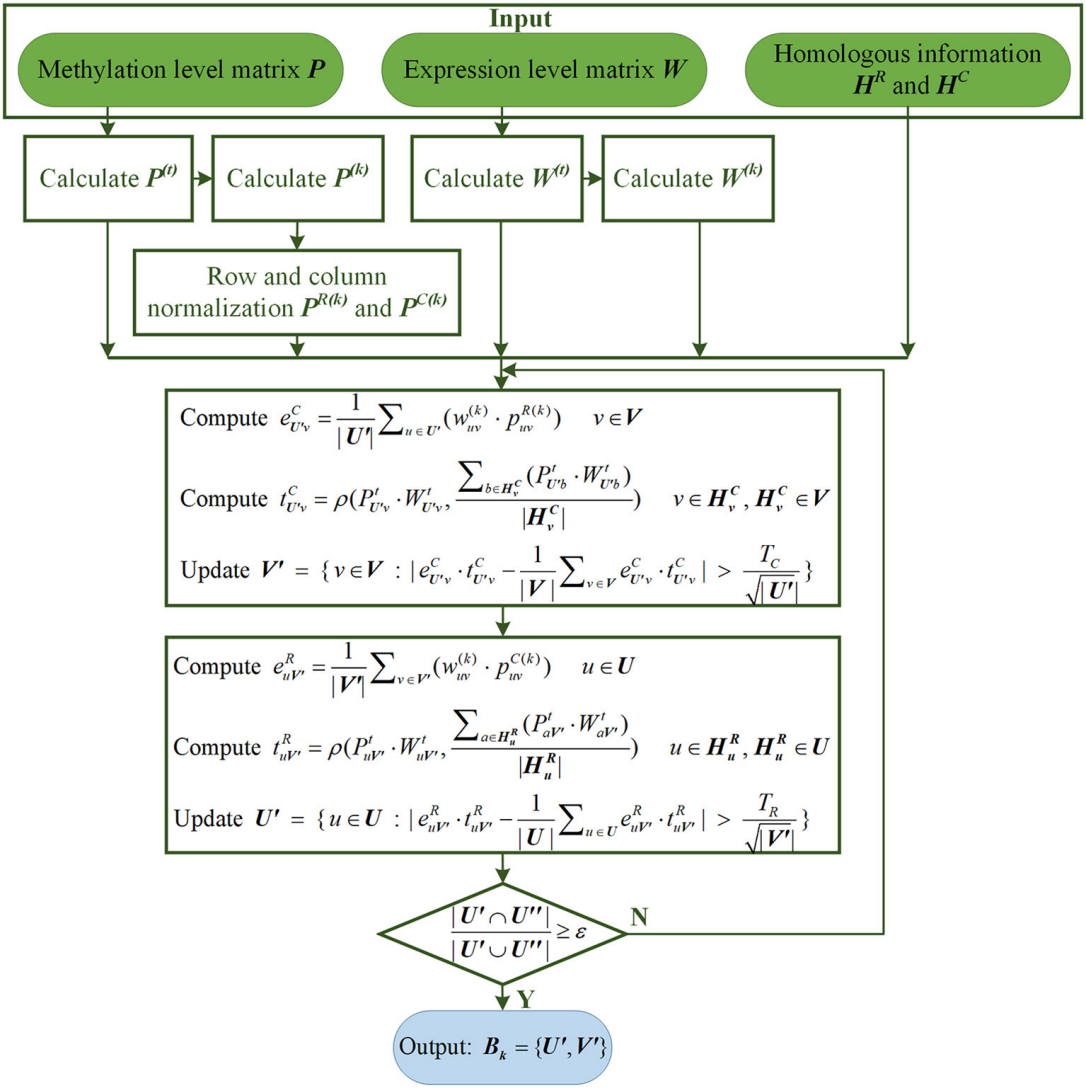
The flow chart of searching for the *k*-th LFB by REW-ISA V2.

Using the preset hyperparameters *T*_*R*_ and *T*_*C*_, ***U*****′** and ***V*****′** are updated iteratively by (12, 13) until convergence is satisfied (or the maximum number of preset iterations is reached). The convergence condition is shown in (14).

(14) $$\begin{array}{l} \frac{\left|U^{\prime }\;\cap \;U^{\prime \prime }\right|}{\left|U^{\prime }\;\cup \;U^{\prime \prime }\right|}\geq \varepsilon \end{array}$$

where ε is the default convergence criteria, and its value is slightly <1. ***U”*** represents the site's subset in the previous iteration, and ***U*****′** represents its subset in the current iteration. If the algorithm converges within the maximum number of iterations, it means that the *k*-th LFB, $B_{k}=\left\{U^{\prime },\;V^{\prime }\right\}$ has been found. The flow chart of searching for the *k*-th LFB by REW-ISA V2 is shown in Figure 1.

Then the algorithm will return to (8) and continue to look for the next LFB. Conversely, if the convergence condition of (14) is not satisfied when the algorithm reaches the maximum number of iterations, REW-ISA V2 will automatically terminate and output all previously obtained LFBs. We recommend setting ε to 0.99 and the maximum number of iterations not <50. The closer the value of ε is to 1 and the greater the maximum number of iterations, the more accurate the LFBs obtained by REW-ISA V2. The REW-ISA V2 algorithm based on R language can be downloaded freely from https://github.com/labiip/REWISAV2.

### Enrichment Constraint Framework

It can be seen from (12, 13) that the selection of *T*_*R*_ and *T*_*C*_ will greatly affect the biological significance of the obtained LFBs. Therefore, based on Meng et al. (2009), we introduced a grid search-based enrichment constraint framework for the algorithm to optimize *T*_*R*_ and *T*_*C*_ selection further. For LFBs obtained under different *T*_*R*_ and *T*_*C*_ combinations, we need to extract the genes corresponding to the m^6^A sites in each LFB and then perform GO analysis based on “clusterProfiler” (Yu et al., 2012) for each LFB. For the range of *T*_*R*_ and *T*_*C*_, we recommend setting it between 0 and 3, and the step size is 0.1. On this basis, the range of specific thresholds should be appropriately adjusted according to the input real data. Assuming that a LFB is obtained, the number of genes corresponding to the m^6^A site contained in it is *M*. The number of GO terms obtained by GO analysis of the LFB is *l*. Then the weighted enrichment score (WE_score) (Li et al., 2012) of this LFB can be calculated by (15).

(15) $$\begin{array}{l} \{\begin{array}{l} s_{i}=-\log\left(p_{i}\right)\\ \text{WE\_}\text{score}=\frac{s_{1}m_{1}/M+s_{2}m_{2}/M+\cdot \cdot \cdot +s_{l}m_{l}/M}{m_{1}/M+m_{2}/M+\cdot \cdot \cdot +m_{l}/M+m_{non}/M}, \end{array} \end{array}$$

where *p*_*i*_ is the *p*-value of the *i*-th GO term, *m*_*i*_ is the number of genes of the *i*-th GO term enriched, *m*_*non*_ is the number of genes covered by LFB but not enriched by any GO term. The higher the WE_score, the stronger the biological significance of this LFB.

However, as the number of genes corresponding to the sites in LFB increases, WE_score will also show an increasing trend, as shown in Supplementary Figure 1. Therefore, only using WE_score to evaluate the biological significance of obtained LFBs is not perfect, and the number of genes corresponding to the sites in LFBs also needs to be considered. Assume that the data analyzed contain a total of *M*_*all*_ genes, and further assume an obtained LFB is containing *M* genes and record its WE_score as *W*_*m*_. We randomly select *M* genes from all genomes, and their WE_score is recorded as *W*_*rm*_. The relative promotion rate (RPR) of WE_score can be further calculated, as shown in (16).

(16) $$\begin{array}{l} \text{RPR}=\frac{M\left(W_{m}-W_{rm}\right)}{M_{all}W_{rm}}. \end{array}$$

The larger the RPR is, the larger the area of the obtained LFB is, and the more biological significance of the obtained LFB is. On the one hand, in the actual process of mining LFBs, we hope to get more LFBs. On the other hand, we hope to get LFBs with rich biological significance. Therefore, the number of LFBs obtained by each pair of threshold combinations is obtained by grid search under different *T*_*R*_ and *T*_*C*_ combinations. The threshold combinations corresponding to the maximum number of LFBs are selected. Then, the average RPR of the LFBs is calculated based on the selected combination of *T*_*R*_ and *T*_*C*_. Finally, the optimal *T*_*R*_ and *T*_*C*_ are the threshold combinations corresponding to the maximum average RPR.

## Results

We collected 32 samples from 10 publicly human m^6^A MeRIP-seq datasets (Dominissini et al., 2012; Meyer et al., 2012; Fustin et al., 2013; Batista et al., 2014; Schwartz et al., 2014; Wang et al., 2014; Barbieri et al., 2017; Li et al., 2017; Pendleton et al., 2017) to mine potential LFBs, most of which can be retrieved from the MeT-DB V2.0 database (Liu et al., 2018). Table 1 summarizes the MeRIP-seq real data set used in this project. Then, calculate the corresponding ***P*** and ***W*** through (1) and (2), and perform REW-ISA V2. Within the range of *T*_*R*_ being 0.1-2 with step size 0.1, and *T*_*C*_ being 0.1–2 with step size 0.1, *T*_*R*_ and *T*_*C*_ are optimized through the enrichment constraint framework. The experiments were repeated ten times for each parameter setting. Although optimizing *T*_*R*_ and *T*_*C*_ based on the gathered biological significance may produce biased results. However, this process provides guidance for the selection of *T*_*R*_ and *T*_*C*_. Finally, under the optimal *T*_*R*_ of 0.4 and the optimal *T*_*C*_ of 0.7, a total of fifteen LFBs are obtained. The number of m^6^A sites, the number of genes corresponding to m^6^A sites and the number of conditions contained in these LFBs are shown in Supplementary Table 1.

**Table 1:** MeRIP-seq datasets used in the study.

**ID**	**GEO accession**	**Cell line**	**Treatment**	**Source**
1–4	SRR456542–SRR456549, SRR456551–SRR456557	HepG2	UV, HGF, IFN, UT	Dominissini et al., 2012
5–6	SRR903368–SRR903379	U2OS	CTL, DAA	Fustin et al., 2013
7–10	SRR847358–SRR847377	HeLa	Ctrl, METTL3-, METTL14-, WTAP-	Liu et al., 2014
11–12	SRR1182582–SRR1182590	ES/NPC	hNPC, hESC	Schwartz et al., 2014
13–18	SRR1182591–SRR1182596, SRR494613–SRR494618, SRR5080301–SRR50312	HEK293	Ctrl, WTAP-, METTL3-, METTL16-	Meyer et al., 2012; Schwartz et al., 2014; Pendleton et al., 2017
19–21	SRR1182597–SRR1182602	OKMS	D0, D5_WITH_DOX, D5_WO_DOX	Schwartz et al., 2014
22–26	SRR1182603–SRR1182630	A549	Ctrl, METTL3-, METTL14-, WTAP-, KIAA1429-	Schwartz et al., 2014
27–28	SRR3066062–SRR3066069	AML	Ctrl, FTO+	Li et al., 2017
29–30	SRR5239086–SRR5239109	AML2	Ctrl, METTL3-	Barbieri et al., 2017
31–32	SRR1035213–SRR1035224	ESC	T0, T48	Batista et al., 2014

**Figure 2:**
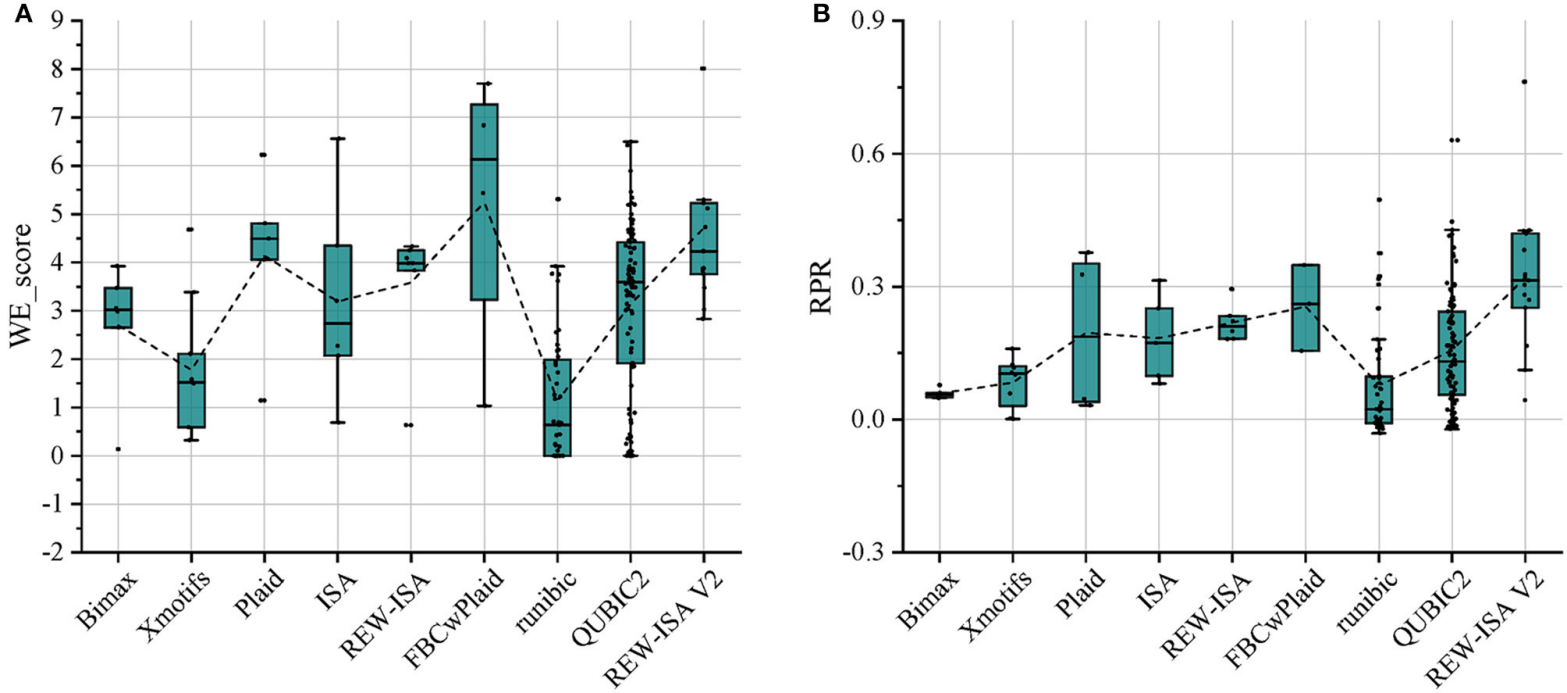
Compare the LFBs obtained by the nine methods through two enrichment evaluation indicators. **(A)** The WE_score comparison of LFBs obtained by each method. **(B)** The RPR comparison of LFBs obtained by each method. The connecting line between the boxes is the mean connecting line.

For the above-mentioned real data set, Bimax (Prelić et al., 2006), Xmotifs (Murali and Kasif, 2003), Plaid (Lazzeroni and Owen, 2002), ISA (Bergmann et al., 2003), REW-ISA (Zhang et al., 2020), FBCwPlaid (Chen et al., 2021), runibic (Orzechowski et al., 2018a), and QUBIC2 (Xie et al., 2020) were all included for comparison with REW-ISA V2. To make the LFBs obtained by the above methods have significant biological significance, the parameters of these methods have been appropriately adjusted. For each LFB obtained by each method, the two enrichment indicators, WE_score and RPR, were both calculated for evaluation. The comparison results are shown in Figures 2A,B, respectively. As can be seen from Figure 2A, the average WE_score of the LFBs obtained by the REW-ISA V2 algorithm is higher than that of ISA and REW-ISA, which indicates that the fusion of homologous information is effective for mining LFBs. Although the average WE_score of LFBs obtained by REW-ISA V2 is lower than that of the FBCwPlaid algorithm, there are significant differences in RPR between the two methods. After further analysis of the LFBs, we found that this was caused by the size of LFBs found by REW-ISA V2 was smaller than that found by the FBCwPlaid algorithm. In other words, the LFBs found by REW-ISA V2 had higher enrichment scores with fewer corresponding genes. Besides, we can find that runbic and QUBIC2 do not perform well in the task of m^6^A hyper-methylation pattern recognition. It may be due to the following two points. On the one hand, the two algorithms mainly identify the trend-preserving biclusters, which is different from the hyper-methylation bicluster. On the other hand, the LFBs obtained are generally small. This also reflects the need of developing biclustering methods for epi-transcriptome data. In a word, the average RPR of LFBs inferred by REW-ISA V2 is significantly higher than that of other biclustering algorithms, which means that the LFBs obtained by REW-ISA V2 may be more biologically significant.

**Figure 3:**
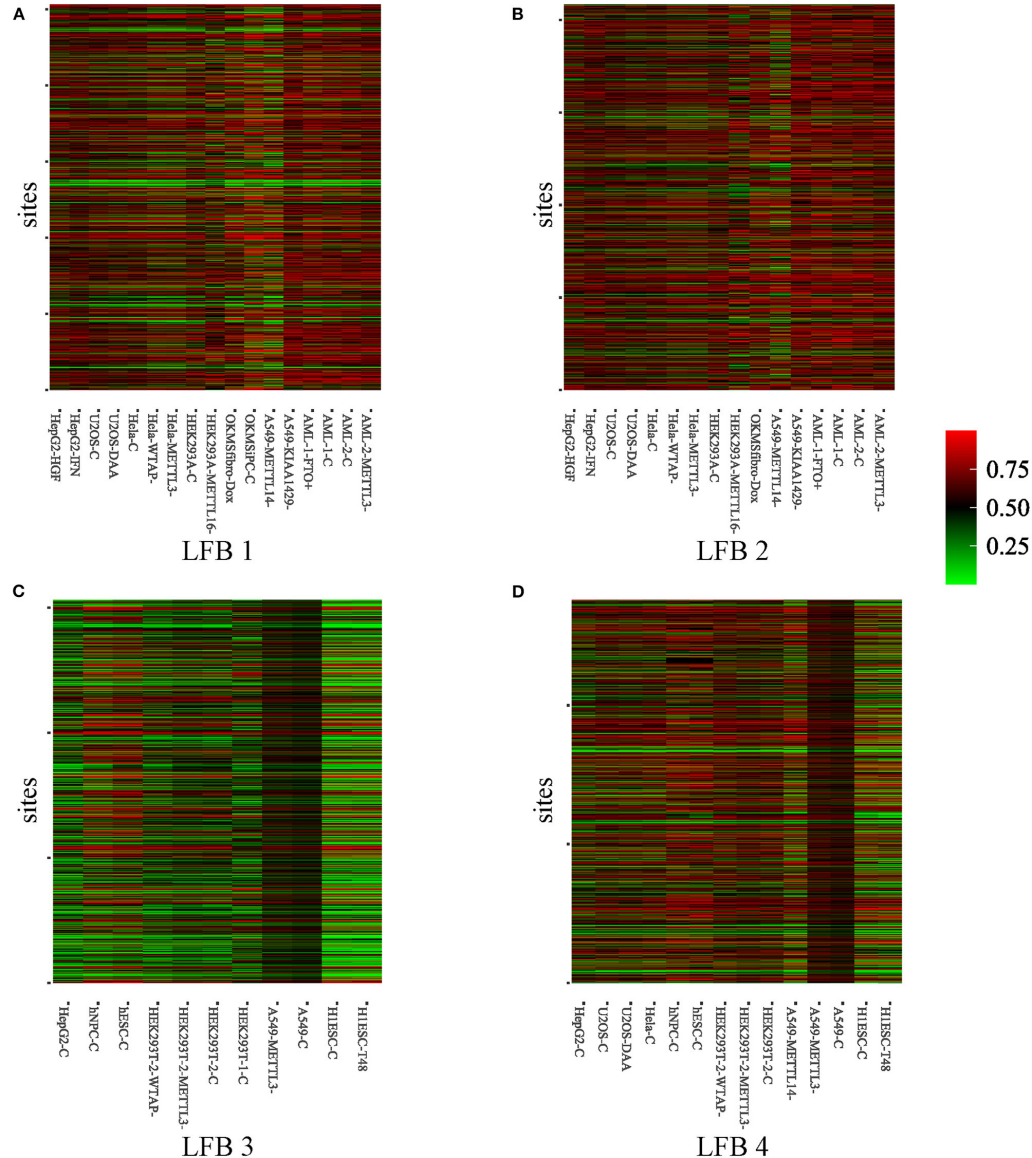
Heatmaps of methylation level of the four selected LFBs. **(A)** The methylation level heatmap of the LFB1. **(B)** The methylation level heatmap of the LFB2. **(C)** The methylation level heatmap of the LFB3. **(D)** The methylation level heatmap of the LFB4.

To further explore the biological significance of the obtained LFBs, we selected four LFBs with more sites from the fifteen LFBs. As can be seen from Supplementary Table 1, for the four selected LFBs, they cover 1,256, 1,619, 824, and 1,148 genes, respectively. An important feature of any biclustering is the identified subsets of conditions, so the conditions contained in the four selected LFBs are explored in detail, as shown in Supplementary Table 2. The methylation level heatmaps of the four selected LFBs are shown in Figure 3. For the KEGG pathway analysis, six KEGG pathways known to be regulated by RNA methylation were selected (Dominissini et al., 2012; Xiang et al., 2017), such as apoptosis, DNA repair, fatty acid metabolism, etc. Then, Fisher's exact test was used to verify whether each LFB was significantly enriched in some specific pathways. The output *p*-value shows the correlation between the four LFBs obtained and six biological pathways, as well as the importance of multiple hypothesis correction. We could see from Supplementary Table 3 that the four selected LFBs are significantly enriched in the ultraviolet (UV) response up. Although the enrichment degree of LFB2 is lower than that of the other three LFBs in the UV response up, its enrichment in the apoptosis is significantly higher than that of the other three LFBs, indicating that LFB2 may further affect apoptosis through some other m^6^A-related pathways. Besides, LFB1, LFB3, and LFB4 are also significantly enriched in DNA repair, which may be related to DNA damage caused by ultraviolet radiation. Since m^6^A has been proved to be related to stem cell differentiation and cancer progression (Batista et al., 2014), there is a reasonable explanation for enriching LFB1 and LFB3 in fatty acid metabolism. As the main components of neutral fat, phospholipids and glycolipids, fatty acids can meet various body needs and regulate metabolism, growth and development (Azain, 2004). The p53 pathway enriched in LFB4 indicates that LFB4 may be related to stress signal, regulation of intracellular homeostasis, chromosome segregation, and cell division (Harris and Levine, 2005). Through the above analysis, it is not difficult to see that the LFBs obtained by REW-ISA V2 have more significant biological significance than the randomly selected LFB. Therefore, an in-depth analysis of the LFBs obtained may help reveal the specific regulatory mechanism of m^6^A.

To check whether the detected LFBs have biological significance, we further conducted the enzymes substrate specificity experiments on the four selected LFBs. Since LFB covers hyper-methylated sites and conditions, the sites and conditions involved in each LFB are more likely to be the target sites of m^6^A methyltransferases. Therefore, we studied the association between each selected LFB and four m^6^A methyltransferases, including METTL3, METTL14, WTAP as well as KIAA1429. For this purpose, 38,845 METTL3 targeted gene sites, 19,099 METTL14 targeted gene sites, 35,144 WTAP targeted gene sites, and 1,784 KIAA1429 targeted gene sites included in the real data were first identified by TREW tool (Liu et al., 2018). After REW-ISA V2, we summarized the distribution of target RNA methylation sites involved in each LFB (Supplementary Table 4). Then, the association between the sites in each selected LFB and m^6^A methyltransferases target sites was further evaluated by Fisher's exact test. The experimental enrichment results are shown in Supplementary Table 5, where *p*-value indicates the significance of association between sites and methyltransferase target sites. The results showed that the sites contained in the four selected LFBs were significantly enriched in the target sites of the four methyltransferases. This means that under specific conditions, the LFBs obtained by REW-ISA V2 were indeed the collaboratively hyper-methylated sites, which will help biologists to further study the specific regulation mechanism of m^6^A. The detailed analysis process and results can be obtained in the Supplementary Materials.

## Discussion

Although more and more studies have shown that the modification of m^6^A in RNA is related to many important biological functions, the specific regulatory mechanism of m^6^A is still unclear. To quickly and effectively predict potential functional m^6^A sites from the epi-transcriptome data, it is important to develop some computational algorithms, which will help us have a more comprehensive understanding of m^6^A-related life processes. Based on REW-ISA, in this article, we developed REW-ISA V2 to better reveal the potential local co-methylation patterns across subsets of conditions. REW-ISA V2 was implemented on the real MeRIP-seq data, and a total of 15 LFBs were obtained. Further comparison and analysis show that, compared with other biclustering algorithms, the LFBs obtained by REW-ISA V2 has more significant biological significance.

REW-ISA V2 could obtain reliable biclustering patterns because of the use of homologous information. More specifically, the sites' methylation levels corresponding to the same gene will show a similar trend with a high probability. Similarly, conditions derived from the same cell line will have similar trends in all sites. Therefore, the rational use of homologous information will help to better mine local co-methylation patterns. Of course, REW-ISA V2 still has some deficiencies that need to be improved in the future. First of all, REW-ISA V2 uses simple multiplication to fuse homologous information, which inevitably introduces noise at the same time. Secondly, because the database on which GO analysis depends is incomplete, the enrichment constraint framework designed is prone to human error. Finally, the enrichment constraint framework built into REW-ISA V2 usually takes a long time. In the future, we will use BSig (Henriques and Madeira, 2018) to better evaluate the obtained LFBs and develop a new computational model to overcome these limitations.

## Data Availability Statement

The original contributions presented in the study are included in the article/Supplementary Material, further inquiries can be directed to the corresponding author/s.

## Author Contributions

LZ and SC built the architecture for REW-ISA V2, designed and implemented the experiments, analyzed the result, and wrote the paper. JM conducted the experiments, analyzed the result, and revised the paper. ZL and HL supervised the project, analyzed the result, and revised the paper. All authors read, critically revised, and approved the final manuscript.

## Conflict of Interest

The authors declare that the research was conducted in the absence of any commercial or financial relationships that could be construed as a potential conflict of interest.
