# Supplementary material for: REW-ISA V2: A Biclustering Method Fusing Homologous Information for Analyzing and Mining Epi-Transcriptome Data
Source: Front Genet. 2021 May 28;12:654820. doi: 10.3389/fgene.2021.654820 (PMC8194299; doi:10.3389/fgene.2021.654820)
Supplement: Supplementary file 1 [file Data_Sheet_1.docx]

Supplementary Material

**Supplementary Table 1 The details of the 15 LFBs obtained by REW-ISA V2.**

| LFB ID | 1 | 2 | 3 | 4 | 5 | 6 | 7 | 8 | 9 |
| --- | --- | --- | --- | --- | --- | --- | --- | --- | --- |
| Sites number | 5067 | 4170 | 3063 | 2759 | 2733 | 2095 | 1689 | 1132 | 866 |
| Genes number | 1256 | 1619 | 824 | 1148 | 1027 | 1022 | 957 | 491 | 543 |
| Conditions number | 17 | 16 | 11 | 14 | 15 | 12 | 15 | 8 | 10 |

| LFB ID | 10 | 11 | 12 | 13 | 14 | 15 |
| --- | --- | --- | --- | --- | --- | --- |
| Sites number | 796 | 790 | 569 | 385 | 101 | 96 |
| Genes number | 418 | 379 | 268 | 234 | 53 | 74 |
| Conditions number | 10 | 7 | 6 | 6 | 3 | 4 |

**Supplementary Table 2 The conditions contained in the four selected LFBs.**

| ID | Conditions |
| --- | --- |
| LFB 1 | HEK293T-2-METTL3-, HEK293T-2-C, HEK293T-1-C, HEK293A-C, HEK293A-METTL16-, A549-METTL14-, A549-METTL3-, A549-C, OKMSfibro-Dox, OKMSfibro-C, OKMSiPC-C, HepG2-HGF,  HepG2-C, hNPC-C, hESC-C, Hela-METTL3-, AML-2-C |
| LFB 2 | HEK293T-2-METTL3-, HEK293T-2-C, HEK293T-1-C, HEK293A-C, HEK293A-METTL16-, A549-METTL3-, A549-METTL14-, A549-C, OKMSfibro-Dox, OKMSfibro-C, OKMSiPC-C, hNPC-C, hESC-C, Hela-METTL3-, AML-2-C, HepG2-C |
| LFB 3 | AML-1-FTO+, AML-1-C, AML-2-METTL3-, Hela-C, Hela-WTAP-, Hela-METTL14-, U2OS-C, U2OS-DAA, H1ESC-C, H1ESC-T48, A549-WTAP- |
| LFB 4 | A549-WTAP-, A549-METTL14, A549-METTL3-, A549-C,  AML-1-FTO+, AML-1-C, Hela-C, Hela-METTL14-, Hela-WTAP-, HepG2-C, U2OS-C, U2OS-DAA, H1ESC-C, H1ESC-T48 |

**Supplementary Table 3 KEGG pathway analysis of four LFBs obtained by REW-ISA V2.**

| ID | Number of sites | Enrichment Statistics | KEGG Pathways | | | | | |
| --- | --- | --- | --- | --- | --- | --- | --- | --- |
|  |  |  | Apoptosis | DNA repair | Fatty Acid Metabolism | p53 Pathway | UV response Down | UV response Up |
| Random LFB | 3700 | OR | 1.0663 | 0.6950 | 0.4789 | 1.0554 | 1.3175 | 1.0073 |
|  |  | *p*-value | 0.7581 | 0.1366 | 0.0098 | 0.7845 | 0.1858 | 1.0000 |
|  |  | FDR | 0.8235 | 0.2314 | 0.0504 | 0.8235 | 0.2787 | 1.0000 |
| LFB1 | 5067 | OR | 1.5024 | 1.7277 | 1.8205 | 1.3851 | 0.6706 | 2.1895 |
|  |  | *p*-value | 0.0949 | **0.0297** | **0.0259** | 0.1389 | 0.2964 | **0.0012** |
|  |  | FDR | 0.1780 | 0.0743 | 0.0707 | 0.2314 | 0.3556 | 0.0175 |
| LFB2 | 4170 | OR | 1.7567 | 1.0705 | 1.4425 | 0.8818 | 1.3378 | 1.7064 |
|  |  | *p*-value | **0.0118** | 0.7960 | 0.1553 | 0.6581 | 0.2274 | **0.0189** |
|  |  | FDR | 0.0504 | 0.8235 | 0.2452 | 0.7594 | 0.3101 | 0.0666 |
| LFB3 | 3063 | OR | 1.7469 | 1.8883 | 2.3651 | 1.3239 | 0.3349 | 2.5247 |
|  |  | *p*-value | **0.0435** | **0.0236** | **0.0036** | 0.2955 | **0.0467** | **0.0006** |
|  |  | FDR | 0.0933 | 0.0707 | 0.0357 | 0.3556 | 0.0934 | 0.0175 |
| LFB4 | 2759 | OR | 1.6659 | 1.9159 | 1.3896 | 1.6879 | 1.4322 | 1.9580 |
|  |  | *p*-value | **0.0417** | **0.0102** | 0.2503 | **0.0200** | 0.2108 | **0.0094** |
|  |  | FDR | 0.0933 | 0.0504 | 0.3265 | 0.0666 | 0.3011 | 0.0504 |

Note: OR stands for odds ratio; Fisher's exact test evaluates *p*-value; FDR is calculated following the BH method (Benjamini and Hochberg, 1995).

**Supplementary Table 4 Number of m^6^A methyltransferase target sites in each selected LFB.**

| ID | Methyltransferase Component | | | |
| --- | --- | --- | --- | --- |
|  | METTL3 | METTL14 | WTAP | KIAA1429 |
| LFB1 | 3087 | 2660 | 3155 | 291 |
| LFB2 | 2784 | 1766 | 2628 | 182 |
| LFB3 | 1569 | 1327 | 1512 | 106 |
| LFB4 | 2003 | 1531 | 1919 | 112 |

**Supplementary Table 5 Enzyme specificity analysis of four LFBs obtained by REW-ISA V2.**

| ID | Number of sites | Enrichment Statistics | Methyltransferase Component | | | |
| --- | --- | --- | --- | --- | --- | --- |
|  |  |  | METTL3 | METTL14 | WTAP | KIAA1429 |
| LFB1 | 5067 | OR | 1.2479 | 3.2227 | 1.6708 | 2.5663 |
|  |  | *p*-value | 8.9E-14 | 0.0E+00 | 3.3E-67 | 1.7E-38 |
|  |  | FDR | 1.3E-13 | 0.0E+00 | 7.5E-67 | 2.7E-38 |
| LFB2 | 4170 | OR | 1.6273 | 2.0320 | 1.7170 | 1.8138 |
|  |  | *p*-value | 6.9E-49 | 2.4E-100 | 4.2E-62 | 4.4E-12 |
|  |  | FDR | 1.2E-48 | 1.3E-99 | 8.5E-62 | 5.8E-12 |
| LFB3 | 3063 | OR | 0.8201 | 2.0909 | 0.9493 | 1.3823 |
|  |  | *p*-value | 9.0E-08 | 2.9E-82 | 1.6E-01 | 2.3E-03 |
|  |  | FDR | 1.1E-07 | 9.1E-82 | 1.6E-01 | 2.5E-03 |
| LFB4 | 2759 | OR | 2.1462 | 3.4857 | 2.3008 | 1.6453 |
|  |  | *p*-value | 1.5E-75 | 1.3E-218 | 3.6E-94 | 2.7E-06 |
|  |  | FDR | 3.9E-75 | 1.0E-217 | 1.4E-93 | 3.1E-06 |

**
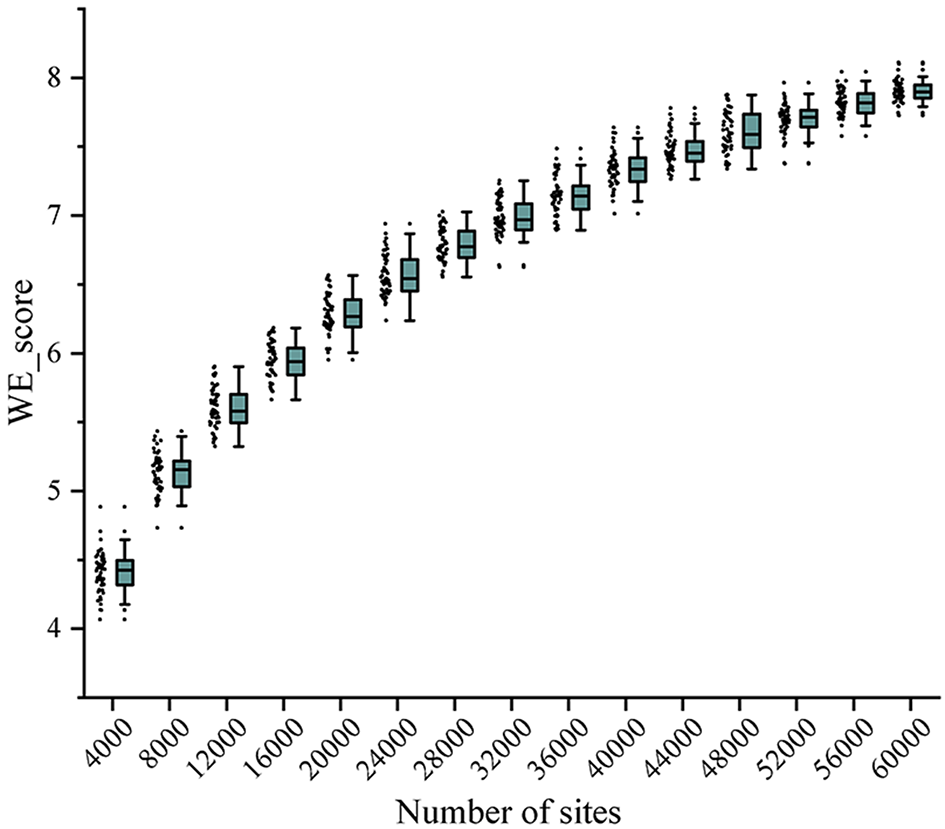
**

**Supplementary Figure 1 The relationship between WE_score and the number of sites. The abscissa coordinate represents the number of randomly selected m^6^A sites, and the vertical coordinate represents the WE_score of genes corresponding to the sites.**
